# The Molting Worm Sheds Its Genetic Secrets

**DOI:** 10.1371/journal.pbio.0030345

**Published:** 2005-08-30

Anyone who winces remembering the challenges of childhood might consider the fate of the developing worm. Four times in its life, a growing nematode worm flips on its side and writhes around to shed its exoskeleton, or cuticle. During each molt, a worm casts aside its cuticle and synthesizes a new protective shell, its primary defense against a harsh environment. Though it's clear that a complex array of signaling proteins and enzymes are required to engineer these rites of passage, only a few genes have been implicated in the process.

Scientists have found critical molting genes in the fruitfly, but most of these are not present in the worm. It may be that the worm has unique molting genes, because its cuticle is more elastic than the hardened casing of an insect. If scientists find molting genes that exist only in the worm, they can begin to unravel the mechanisms that govern these critical phases of a worm's life. They can also develop treatments that target the worm's parasitic cousins—which wreak havoc on humans, livestock, and plants—without producing harmful side effects. In a new study, Alison Frand, Sascha Russel, and Gary Ruvkun searched the entire genome of the worm *Caenorhabditis elegans* for molting genes and identified 159 candidates, using a technique called RNA interference.

In RNA interference, researchers use double-stranded RNA (dsRNA) to block the expression of, or silence, a specific gene by destroying the gene's messenger RNA transcript before it can be translated into protein. These dsRNAs can be expressed in bacteria—a staple food for lab worms—which then multiply into large colonies expressing the same dsRNA. Using a pre-existing “library” of bacterial clones that each express a particular dsRNA, the authors fed groups of larvae one bacterial clone at a time—until thousands of larvae had eaten bacteria with dsRNA designed to silence nearly every one of the worm's 19,427 genes.

After the larvae ate the bacterial clones, the authors screened them for molting defects—which is how they identified the 159 genes. Molting defects mostly left larvae trapped in their old cuticle; those that managed to escape often failed again during the next molt. The majority of candidate molting genes appear to play a role in all four molts, the authors argue, since their inactivation foils molting at several stages. And, significantly, the majority of genes—many of which are found only in worms—exist in worm parasites that infect humans, animals, and plants.

Among the genes identified, the authors found several transcription factors (proteins that activate genes), indicating that molting requires “extensive changes in gene expression.” Other genes are associated with signaling proteins that likely coordinate the activity of different cell types during molting, and many genes code for proteins that are required for protein synthesis—likely to build the new cuticle. Still other genes may help remodel the cuticle.[Fig pbio-0030345-g001]

**Figure pbio-0030345-g001:**
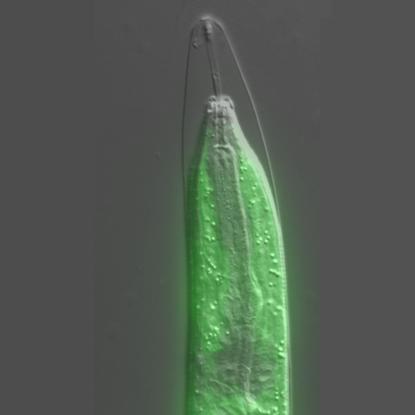
As this Caenorhabditis elegans larva molts between developmental stages, green fluorescent protein allows researchers to trace the expression of one of its molting genes (*mlt-11*). Defects in this gene trap larvae in their cuticle

To monitor the expression of some of these genes and infer their function, the authors tagged a subset of genes—representing many of the functional categories found in the screen—with green fluorescent protein. Since green fluorescent protein glows when a gene is activated, the researchers can see where and when genes are expressed. Fluorescence levels were high just before each molt and dropped off soon after. All of these genes were expressed in the epithelial cells that secrete new cuticle. These results provide strong evidence for the genes' role in molting, since they were expressed both at the right time and the right place. These experiments also allowed Frand et al. to propose a model describing the timing and order of gene expression during molting.

With all the genes identified in this screen, researchers can now start to piece together the overlapping pathways that guide the worm through its formative years. And with the discovery of worm-specific genes, it's likely that more effective treatments await patients with elephantiasis, African river blindness, and other diseases caused by pathogenic nematodes. —*Liza Gross*

